# Do Hispanic Puerto Rican men have worse outcomes after radical prostatectomy? Results from SEARCH

**DOI:** 10.1002/cam4.7012

**Published:** 2024-03-08

**Authors:** Lourdes Guerrios‐Rivera, Jessica L. Janes, Amanda M. De Hoedt, Zachary Klaassen, Martha K. Terris, Matthew R. Cooperberg, Christopher L. Amling, Christopher J. Kane, William J. Aronson, Jay H. Fowke, Stephen J. Freedland

**Affiliations:** ^1^ Urology Section, Surgery Department Veterans Administration Caribbean Healthcare System San Juan Puerto Rico; ^2^ University of Puerto Rico, Medical Sciences Campus San Juan Puerto Rico; ^3^ Section of Urology, Division of Surgery Durham VA Health Care System Durham North Carolina USA; ^4^ Department of Surgery, Section of Urology Augusta University – Medical College of Georgia Augusta Georgia USA; ^5^ Charlie Norwood VA Medical Center Augusta Georgia USA; ^6^ Department of Urology Diller Family Comprehensive Cancer Center, UCSF Helen San Francisco California USA; ^7^ Department of Urology Oregon Health and Science University School of Medicine Portland Oregon USA; ^8^ Department of Urology UC San Diego Health System San Diego California USA; ^9^ VA San Diego Healthcare System San Diego California USA; ^10^ Department of Urology UCLA Medical Center Los Angeles California USA; ^11^ Wadsworth VA Medical Center Los Angeles California USA; ^12^ Division of Epidemiology, Department of Medicine Vanderbilt University Medical Center Nashville Tennessee USA; ^13^ Division of Epidemiology, Department of Preventive Medicine University of Tennessee Health Science Center Memphis Tennessee USA; ^14^ Center for Integrated Research in Cancer and Lifestyle, Division of Urology, Department of Surgery Samuel Oschin Comprehensive Cancer Institute, Cedars‐Sinai Medical Center Los Angeles California USA

## Abstract

**Background:**

We previously reported that outcomes after radical prostatectomy (RP) were similar among non‐Hispanic Black, non‐Hispanic White, and Hispanic White Veterans Affairs (VA) patients. However, prostate cancer (PC) mortality in Puerto Rican Hispanics (PRH) may be higher than in other Hispanic groups. Data focused on PRH patients is sparse; thus, we tested the association between PR ethnicity and outcomes after RP.

**Methods:**

Analysis included men in SEARCH cohort who underwent RP (1988–2020, *n* = 8311). PRH patients (*n* = 642) were treated at the PR VA, and outcomes were compared to patients treated in the Continental US regardless of race. Logistic regression was used to test the associations between PRH and PC aggressiveness, adjusting for demographic and clinicopathological features. Multivariable Cox models were used to investigate PRH versus Continental differences in biochemical recurrence (BCR), metastases, castration‐resistant PC (CRPC), and PC‐specific mortality (PCSM).

**Results:**

Compared to Continental patients, PRH patients had lower adjusted odds of pathological grade group ≥2 (*p* < 0.001), lymph node metastasis (*p* < 0.001), and positive margins (*p* < 0.001). In contrast, PRH patients had higher odds of extracapsular extension (*p* < 0.001). In Cox models, PRH patients had a higher risk for BCR (HR = 1.27, *p* < 0.001), metastases (HR = 1.49, *p* = 0.014), CRPC (HR = 1.80, *p* = 0.001), and PCSM (HR = 1.74, *p* = 0.011). Further adjustment for extracapsular extension and other pathological variables strengthened these findings.

**Conclusions:**

In an equal access setting, PRH RP patients generally had better pathological features, but despite this, they had significantly worse post‐treatment outcomes than men from the Continental US, regardless of race. The reasons for the poorer prognosis among PRH men require further research.

## Graphical abstract

In an equal access medical services setting, Puerto Rican men after radical prostatectomy surgery generally had better pathological features, but despite this, they had significantly worse posttreatment outcomes than men from the Continental US.

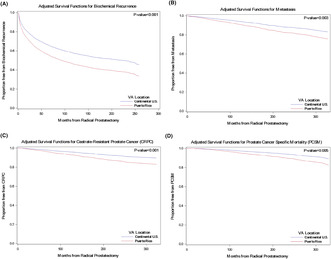

## 1 INTRODUCTION

The majority of prior studies investigating race/ethnicity differences in PC outcomes have focused on Black versus non‐Hispanic White (NHW) cohorts.^1^ Few studies have focused on PC outcomes among Hispanic men. Those that did often found that Hispanics, when analyzed in the aggregate, have similar PC outcomes to NHWs.^2^ Indeed, we previously studied outcomes among an aggregated group of Hispanic men in the Shared Equal Access Regional Cancer Hospital (SEARCH) Database,^3^ a multicenter cohort derived from the VA healthcare system, and found no PC outcome differences between Black, NHW, and Hispanic men. However, such analyses that combine all Hispanic communities ignore the range of biological and cultural diversity across Hispanic populations that may impact PC. Indeed, beliefs and perceptions regarding patient care may differ across groups, creating barriers that impact outcomes and contributing to PC disparities within and between Hispanics and Black men.^4^ Recently, substantial differences in PC outcomes have been described across Hispanic communities, with a worse overall PC survival among Puerto Ricans compared to Mexican men or many other Hispanic subgroups.^5^, ^6^ Furthermore, Puerto Rican men have a higher PC mortality compared to NHW or Hispanic men living in the continental U.S.^7^ Indeed, PC mortality among Puerto Rican Hispanic (PRH) men is second only to Black men in the U.S.^6^, ^7^

Prostate cancer progression and prognosis in Puerto Rico remain largely uninvestigated. It is not understood whether the higher mortality rates in Puerto Rico reflect higher incidence rates of advanced cancers, undertreatment, treatment resistance, poorer follow‐up care, or a combination of effects. Our hypothesis was that PRH patients would have a higher prevalence of adverse pathological features compared to Black and NHW men. Using the SEARCH cohort, we examined whether adverse pathological features will be more prevalent in PRH patients and whether these adverse features in PRH will contribute to higher rates of biochemical recurrence (BCR), lymph node metastasis, castrate‐resistant prostate cancer (CRPC), and PC mortality among PRH patients compared with men treated in the Continental US.

## 2 MATERIALS AND METHODS

### 2.1 Patient Population

The SEARCH cohort captures clinical data from all men with PC treated with radical prostatectomy (RP) at nine Veterans Affairs hospitals nationwide.^8^ After obtaining institutional review board approval (IRB No. 1827) at the Durham VA, data on patients who underwent RP between 1988 and 2020 at Veterans Affairs hospitals in West Los Angeles, San Diego, San Francisco, and Palo Alto, California; Augusta, Georgia; Portland, Oregon; Asheville, Durham, North Carolina; and San Juan, Puerto Rico, were entered in the SEARCH database. Among the total 9931 SEARCH patients, patients who had undergone neoadjuvant hormonal therapy or had prior radiation were excluded from the study. We also excluded those with missing variables of interest, leaving a total of 8311 patients for our analysis.

Of these 8311 patients, 642 (8%) were treated at the Veterans Affairs Caribbean Healthcare System (VACHS) located in San Juan, Puerto Rico, and were categorized as Puerto Rican Hispanic (PRH) patients regardless of race. Men treated at another VA hospital were coded as Continental US. We previously did not find any difference in PC outcomes between Continental Hispanic, Black, or NHW patients in SEARCH,^3^ and thus we grouped all SEARCH patients in the Continental US group regardless of race/ethnicity as “Continental”.

### 2.2 Study variables

The primary covariate was VA location (PRH versus Continental US). Age, race, and BMI were abstracted from the medical record at the time of RP. Preoperative clinical characteristics included biopsy grade group, prostate‐specific antigen (PSA) (ng/mL), and clinical stage. Postoperative pathological characteristics included pathological grade group, extracapsular extension, seminal vesicle invasion, surgical margins, and lymph node metastasis and were included as covariates in primary models.

The primary outcome was the time to prostate cancer‐specific mortality (PCSM). Secondary outcomes included time to biochemical recurrence (BCR), time to castrate‐resistant prostate cancer (CRPC), time to metastases, and adverse pathology features at diagnosis (e.g., Grade Group ≥2, extracapsular extension, etc.). PCSM was defined as death with metastatic progressive CRPC, without another obvious cause of death. BCR was defined as a single PSA >0.2 ng/mL, two concentrations at 0.2 ng/mL, or salvage treatment for an elevated postoperative PSA. Patients who received radiation for undetectable PSA were considered to have adjuvant radiation and were censored for BCR. CRPC was defined as a PSA increase of ≥2 ng/mL and ≥ 25% from the post‐androgen deprivation therapy PSA nadir (the lowest PSA achieved after ADT) while being castrate, defined as serum testosterone <50 ng/dL, bilateral orchiectomy, or continuous receipt of luteinizing hormone releasing hormone agonist or antagonist.^9^ Metastases were typically identified by bone scan or computer tomography (CT) imaging performed as per attending physician's discretion and assessed by trained personnel to determine the development of metastases.

### 2.3 Statistical analysis

The distributions of baseline demographic and clinical features were compared between patients treated at Puerto Rico and Continental US using Wilcoxon rank‐sum tests for continuous variables and chi‐squared tests for categorical variables. Multivariable logistic regression models were used to evaluate differences in adverse pathological features between Puerto Rico and Continental US patients, adjusting for year of surgery, age at surgery, race, and preoperative clinical features (PSA, stage, and biopsy grade group). The model for lymph node metastasis was limited to patients who had lymph node dissection performed (5345 Continental US patients & 530 PRH patients). Standard crude Kaplan–Meier curves are included as supplemental material. Univariable and multivariable Cox proportional hazards regression models were used to evaluate differences in time from RP to PCSM and other time‐to‐event outcomes between Puerto Rico and Continental US patients. As has been done in prior analyses of SEARCH.^10^ Two sets of multivariable models were used: (1) a “pre‐operative” model that adjusted for demographic and clinical features (age, year of surgery, race, PSA, clinical stage, and biopsy grade) and (2) a “postoperative” model that adjusted for demographic and pathological features (age, year of surgery, race, PSA, pathological grade, positive surgical margins, seminal vesicle invasion, lymph node metastasis, and extracapsular extension). Survival functions estimated from these final, postoperative multivariable models were plotted and stratified by VA location (PRH vs. Continental US).

To assess whether the impact of VA location (PRH vs. Continental US) on PC outcomes depended on various clinical features, interaction terms between VA location and biopsy grade, clinical stage, and year of surgery were tested, and model estimates for VA location were stratified by those factors. For this analysis, year of surgery was categorized as <1994, 1994–2011, and ≥ 2012 to reflect the introduction of PSA in 1994 and recommendation against widespread screening with PSA in 2012 that caused stage migration phenomena. These analyses were limited by small cell sizes in some instances (which can produce potentially biased estimates) and an increased Type 1 error rate due to additional tests and should be interpreted with caution. Nonetheless, these stratified results are presented as supplemental material for interested readers. Statistical significance was set at *p*‐value <0.05. All analyses were performed using SAS 9.4 (Cary, North Carolina).

## 3 RESULTS

### 3.1 Study population & clinical features

The study population included 642 patients treated in Puerto Rico and 7669 patients treated in the Continental US. Compared to Continental US patients, PRH patients were significantly older (62.8 vs. 62.3 years, *p* = 0.032) and had a lower BMI (27.9 vs. 28.6, *p* < 0.001), although these differences were slight (Table 1). A lower proportion of patients were Black in Puerto Rico than in the Continental US (14% vs. 31%, *p* < 0.001). PRH patients had significantly lower pre‐op grade than Continental US patients (e.g., 55% vs. 38% with grade 1, *p* < 0.001). PSA levels did not differ significantly between PRH patients and Continental US patients, nor did clinical stage (Table 1).

**TABLE 1:** Clinical characteristics of men who underwent a radical prostatectomy.

	Continental U.S. (*N* = 7669)	Puerto Rico (*N* = 642)	*p* Value
Age at surgery			0.0324
Mean (SD)	62.3 (6.2)	62.8 (5.8)	
Median	63.0	64.0	
Q1, Q3	58.0, 67.0	59.0, 67.0	
Year of surgery			0.7325
Mean (SD)	2007.6 (7.3)	2007.8 (6.2)	
Median	2009.0	2008.0	
Q1, Q3	2002.0, 2013.0	2004.0, 2013.0	
Year of surgery			<0.0001
<1994	260 (3%)	5 (1%)	
1994–2011	4705 (61%)	449 (70%)	
>=2012	2704 (35%)	188 (29%)	
BMI at surgery			0.0011
Missing	538	49	
Mean (SD)	28.6 (4.7)	27.9 (4.1)	
Median	28.2	27.7	
Q1, Q3	25.4, 31.3	25.0, 30.4	
PSA (ng/mL) at surgery			0.2792
Mean (SD)	9.0 (9.2)	8.4 (7.8)	
Median	6.6	6.6	
Q1, Q3	4.8, 10.0	4.8, 9.1	
Race			<0.0001
White	5017 (65%)	543 (85%)	
Black	2406 (31%)	88 (14%)	
Other	246 (3%)	11 (2%)	
Pre‐op Grade			<0.0001
1	2927 (38%)	350 (55%)	
2	2330 (30%)	187 (29%)	
3	1051 (14%)	44 (7%)	
4–5	1361 (18%)	61 (10%)	
Clinical stage			0.1035
T1	4232 (55%)	327 (51%)	
T2	3363 (44%)	307 (48%)	
T3/T4	74 (1%)	8 (1%)	
Follow‐up			0.0001
Mean (SD)	119.5 (70.1)	128.5 (65.8)	
Median	110.9	125.6	
Q1, Q3	64.5, 167.9	77.8, 174.9	
Adjuvant therapy			0.0206
No	7392 (96%)	630 (98%)	
Yes	277 (4%)	12 (2%)	
Pelvic lymph node dissection done?			<0.0001
No	2324 (30%)	112 (17%)	
Yes	5345 (70%)	530 (83%)	

### 3.2 Adverse pathological features

Compared to the Continental US, PRH patients had significantly lower proportions with pathological grade ≥2, seminal vesicle invasion, positive surgical margins, and lymph node involvement (Table 2). In contrast, PRH patients had a significantly higher proportion with extracapsular extension compared to the Continental US patients.

**TABLE 2:** Odds of adverse pathological features for Puerto Rico patients compared to Continental U.S. patients estimated from multivariable logistic regression models (*N* = 8311).

Outcome	VA Location	Event/Total (%)	OR^a^ (95% CI)	*p* Value
Post‐op Grade ≥2	Continental US	5753/7669 (75%)	Ref.	
	Puerto Rico	380/642 (59%)	0.52 (0.43, 0.63)	<0.001
Lymph node metastasis^b^	Continental US	303/5345 (6%)	Ref.	
	Puerto Rico	6/530 (1%)	0.25 (0.11, 0.57)	<0.001
Positive surgical margins	Continental US	2901/7669 (38%)	Ref.	
	Puerto Rico	125/642 (19%)	0.43 (0.35, 0.53)	<0.001
Seminal Vesicle Invasion	Continental US	886/7669 (12%)	Ref.	
	Puerto Rico	45/642 (7%)	0.75 (0.54, 1.04)	0.089
Extracapsular extension	Continental US	1866/7669 (24%)	Ref.	
	Puerto Rico	189/642 (29%)	1.55 (1.28, 1.87)	<0.001

Abbreviations: CI, confidence interval; OR, odds ratio.

^a^ Logistic regression models adjusted for VA location, biopsy Gleason grade, pre‐operative PSA, year of surgery, age at surgery, race, and clinical stage.

^b^ Model for lymph node metastasis only among patients who had lymph node dissection done.

Multivariable odds ratios (OR) and 95% confidence intervals (CI) for adverse pathological features are displayed for VA location (PRH vs. Continental US) in Table 2. Estimates for all covariates in the multivariable model are summarized in Table S1. After adjusting for demographics, biopsy grade, and clinical factors, PRH patients had significantly lower odds of a pathological grade group ≥2 (*p* < 0.001), lymph node metastasis (*p* < 0.001), and positive surgical margins (*p* < 0.001) compared to Continental US patients (Table 2 and Table S1). No significant differences were observed in seminal vesicle invasion between PRH and Continental US patients (*p* = 0.089), while PRH patients had significantly higher odds of extracapsular extension (*p* < 0.001) on multivariable analysis.

Significant interactions were observed between VA location and biopsy grade for positive surgical margins and extracapsular extension, and between VA location and year of surgery for the same two outcomes (all *p* < 0.001; Table S3). Overall, PRH patients with low‐grade tumors or treated in earlier years were less likely to have positive margins than Continental US patients. In contrast, PRH patients with low‐grade tumors or treated in earlier years were more likely to have extracapsular extension. Other interactions were nonsignificant, although sample size for these analyses was more limited, as evident in Table 1.

### 3.3 Prostate Cancer Outcomes

**FIGURE 1:**
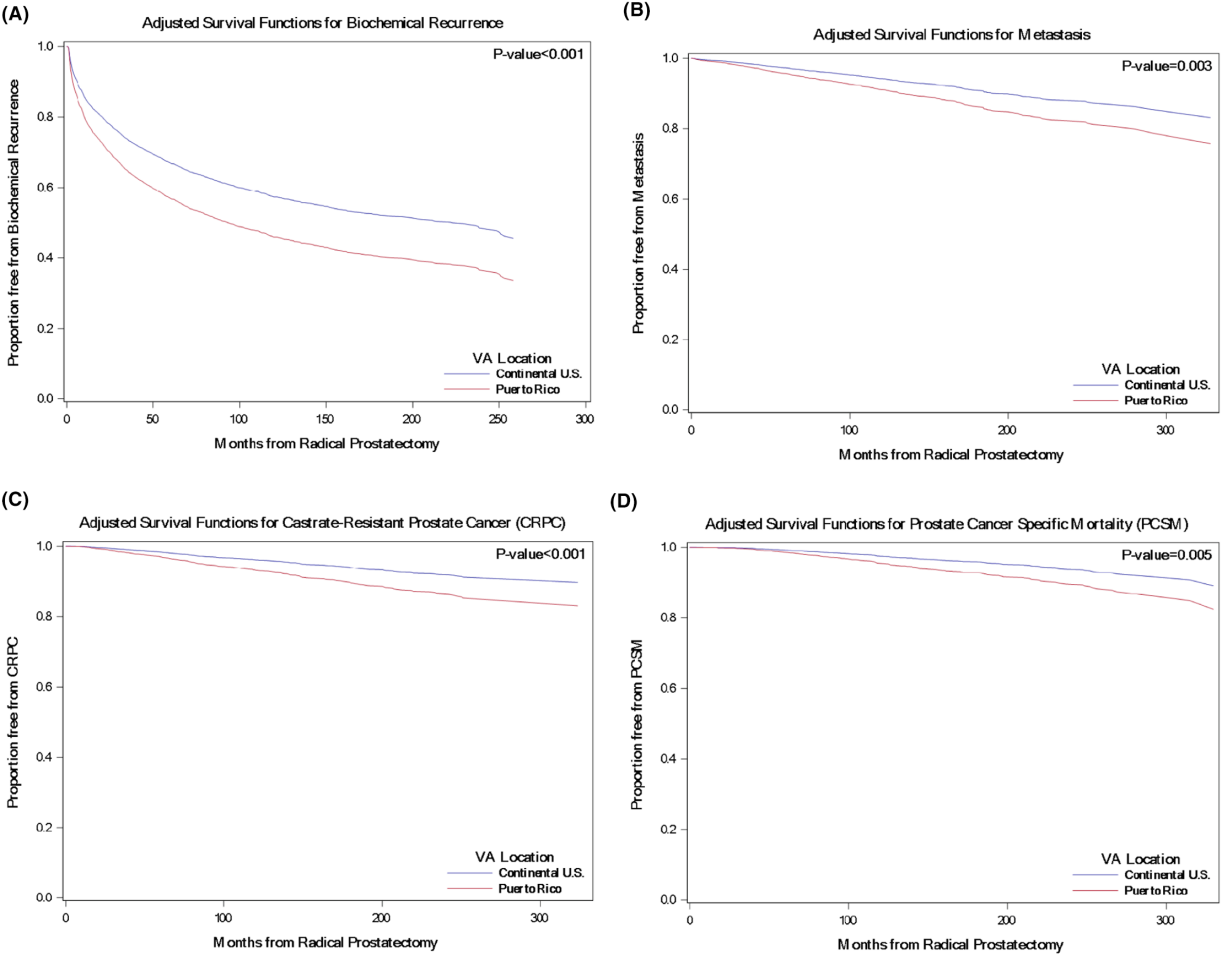
Multivariable survival curve for (A) biochemical recurrence (BCR), (B) metastasis, (C) castrate‐resistant prostate cancer (CRPC), (D) prostate cancer specific mortality (PCSM) stratified by VA location adjusted for postoperative characteristics (*N* = 8311).

The median duration of follow‐up was significantly longer for Puerto Rico (125.6 months) than for Continental US (110.9 months) patients (*p* < 0.001) (Table 1). Among Continental US patients, 215 (3%) died of PC compared to 24 (4%) of PRH patients (Table 3). Hazard ratios (HR) and 95% confidence intervals (CI) are displayed for VA location (PRH vs. Continental US) across crude and adjusted models in Table 3. Table S2 contains results from the full postoperative multivariable model. On the crude analysis, there was a non‐significantly higher risk of PCSM in PRH patients compared to Continental US patients (HR = 1.23, *p* = 0.33) (Table 3). However, after adjusting for relevant clinical and demographic characteristics, PCSM was significantly higher in PRH patients compared to the Continental US patients (HR = 1.74, *p* = 0.01). Further adjustment for pathological features, including grade, extracapsular extension, surgical margins, and seminal vesicle invasion, increased the strength of the positive association between PRH and PCSM (HR = 1.89, *p* = 0.005; Figure 1D and Table S2).

**TABLE 3:** Risk of long‐term PC outcomes for Puerto Rico patients compared to Continental U.S. patients estimated from univariable and multivariable Cox proportional hazards models (*N* = 8311).

	VA Location	Event/Total	Crude Model		Multivariable Model 1^a^		Multivariable Model 2^b^	
			HR (95% CI)	*p* Value	HR (95% CI)	*p* Value	HR (95% CI)	*p* Value
PCSM	Continental US	215/7669	Ref.		Ref.		Ref.	
	Puerto Rico	24/642	1.23 (0.81, 1.88)	0.333	1.74 (1.13, 2.68)	0.011	1.89 (1.21, 2.95)	0.005
Metastases	Continental US	439/7669	Ref.		Ref.		Ref.	
	Puerto Rico	44/642	1.11 (0.82, 1.52)	0.498	1.49 (1.09, 2.03)	0.014	1.64 (1.18, 2.26)	0.003
CRPC	Continental US	308/7669	Ref.		Ref.		Ref.	
	Puerto Rico	35/642	1.26 (0.89, 1.79)	0.193	1.80 (1.26, 2.56)	0.001	1.90 (1.32, 2.75)	<0.001
BCR	Continental US	2774/7669	Ref.		Ref.		Ref.	
	Puerto Rico	268/642	1.07 (0.94, 1.21)	0.288	1.27 (1.12, 1.44)	<0.001	1.51 (1.33, 1.72)	<0.001

Abbreviations: BCR, Biochemical Recurrence; CI, Confidence Interval; CRPC, Castrate‐Resistant Prostate Cancer; HR, Hazard ratio; PCSM, Prostate Cancer‐Specific Mortality;

^a^ Model adjusted for the following clinical characteristics: VA location, biopsy Gleason grade, pre‐operative PSA, year of surgery, age at surgery, race, and clinical stage.

^b^ Model adjusted for the following pathological features: VA location, pathological Gleason grade, pre‐operative PSA, year of surgery, age at surgery, race, extracapsular extension, seminal vesicle invasion, surgical margins, and lymph node metastasis.

Secondary outcomes included metastasis, CRPC, and BCR. A total of 439 (6%) Continental US patients developed metastases compared to 44 (7%) PRH patients. For CRPC, 308 (4%) Continental US patients became castrate‐resistant compared to 35 (5%) PRH patients. Finally, 2774 (36%) Continental US patients had biochemical recurrence following RP compared to 268 (42%) PRH patients (Table 3). Similar to PCSM, the risk of metastases (Figure 1B), CRPC (Figure 1C), and BCR (Figure 1A) was significantly greater for PRH than Continental US after adjusting for clinical and demographic features only (Table 3) and strengthened when adjusting for pathological features (Table 3 and Table S2).

The only significant interaction between VA location and adverse pathology was with biopsy grade for time to metastasis (*p* = 0.042; Table S4). This finding should be interpreted with caution given only 44 PRH patients with biopsy grade 3.

## 4 DISCUSSION

Few PC outcomes studies have focused on Hispanic men, and often these studies aggregated men from Spanish‐speaking countries into one Hispanic group, ignoring differences between countries of origin.^2^ We studied the association between PR ethnicity and outcomes after RP as definitive treatment for localized PC in an equal access health system. Compared to patients treated in the Continental US, PRH patients generally had tumor pathological features consistent with a better prognosis, including lower levels of high‐grade disease, lymph node metastasis, and positive surgical margins. In contrast, extracapsular extension was significantly more prevalent in PRH patients. After controlling for differences in pathological features, PSA, and demographic factors, PRH patients had significantly worse outcomes following surgery, including an increased risk for BCR, metastasis, and PCSM. These findings imply the need to further elucidate and understand the existing PC disparity in PRH men, as distinct from other Hispanic and non‐Hispanic communities.

It remains unclear why PC mortality among PRH men is higher than that of NHW men and also higher among Hispanic men living in the continental US.^7^ Increased PSA testing in PR may explain observed ethnicity differences in incidence,^11^ but it does not clearly explain higher mortality. In our study, PSA levels in PR and the Continental US were similar, and models controlled for differences in age at diagnosis. To control for possible differences in access to healthcare or treatment as driving a survivorship disadvantage among PR men, we focused on men who were treated within an equal‐access healthcare setting. While multiple prior studies using the SEARCH cohort have explored differences by race comparing non‐Hispanic Blacks versus Whites,^12^, ^13^ no prior study from this cohort has examined outcomes among Puerto Rican Hispanics. Our study is the first to show the existing PC disparity mortality in PR through an equal access healthcare system.

We hypothesized that greater rates of adverse pathology among PRH patients, including extracapsular extension, would account for at least a part of any observed difference in PC outcomes. However, we found that PRH patients had many prognostic markers that would predict a better prognosis than patients in the Continental US. When controlling for factors at diagnosis, such as age, PSA levels, year of surgery, race, and clinical stage, PRH had a higher risk of PCSM (HR = 1.74). Additional control for differences in extracapsular extension, surgical margin status, and other pathological features increased but did not decrease the strength of the association between PRH and higher PCSM (HR = 1.89). Thus, we propose that factors other than tumor diagnostic parameters contribute to PC outcomes in PRH patients.

There have been relatively few prior studies that targeted Hispanic ethnicity and outcomes after definitive treatment for PC with conflicting results.^14^, ^15^, ^16^, ^17^ Aligned with our current findings, Dobbs et al.,^5^ showed that Cubans and Puerto Ricans had worse outcomes compared to other Hispanic sub‐groups. Similarly, results of another recent population‐based study showed higher rates of PCSM after RP for Puerto Rican men than for both NHW men and non‐Hispanic Black men.^17^ The latter findings are interesting in that in Puerto Rico, most men labeled as Hispanic White may have a higher level of genetic African ancestry due to extensive genetic admixture of African, Indigenous, and European populations in PR compared to Hispanic White men in the Continental US.^18^, ^19^ For example, genetic variation at 8q24 is highly enriched for both African ancestry and PC risk, and increased 8q24 variation among PR men may be associated with more aggressive PC in PR.^20^ Furthermore, oncological outcomes in island populations may potentially be impacted due to the level of inbreeding in Caribbean Hispanic families.^21^ Besides, recurrent founder mutations in BRCA1 and 2 genes have been reported in the majority of hereditary breast and ovarian cancer syndrome in Puerto Rico,^22^ therefore recognizing the possible repercussions in the genetic risks of PR men. Collectively, past genomic studies have struggled to include a well‐characterized Hispanic population,^23^ missing the opportunity to identify targetable genomic alterations associated with PC.

Furthermore, lifestyle and cultural factors may come into play in PR. Obesity is associated with an increased risk of PC‐aggressive disease^24^, ^25^ and 77% of the Puerto Rican urban population is overweight or obese.,^26^ compared with 73.6% in the Continental US.^27^ Moreover, obesity is a risk factor for diabetes, and our group recently observed obesity‐related differences in the associations between diabetes and PC outcomes.^28^ If we consider that PR has the highest self‐reported prevalence of DM among all states and territories,^29^ it is plausible that both higher rates of diabetes and obesity may contribute to the worse PC mortality in PR, although this requires formal testing. Altogether, the complex interplay between biology, exposures, and social factors has not yet been elucidated and needs to be addressed. As such, whether the findings of worse outcomes among men from Puerto Rico are due to African ancestry, which is known to impact poor outcomes, or a unique mix of genetics with environmental factors specific to Puerto Rico, it is not known. It is critical to investigate health disparities in a more complete framework that integrates not only the biological dimension but also the social context to start to understand the mechanisms involved.^30^

Our study is not devoid of limitations. First, we focused on men with early‐stage disease treated aggressively via surgery to minimize differences in practice patterns. It is important to note that our study population excluded those with invasive disease as they would not be eligible for prostatectomy, and taking into account our low number of clinical stage T3/T4 cases, our findings for locally extensive disease cases should be interpreted with caution. Therefore, the analysis of PRH patients who present with metastatic disease or increased comorbidities that are not surgical candidates at diagnosis would be an important next step. Second, selective loss to follow‐up, such that otherwise healthier PRH patients are lost, may add selection bias to our results. The VA health system was chosen to minimize differences in access to care and create standardized follow‐up protocols, and the somewhat longer follow‐up period in PR also argues against this possibility. Third, there was no centralized pathological review of the RP specimens in this large multicentered study. Nevertheless, differences in patient care among the VA centers were minimized by controlling for the center in the multivariable analyses. Fourth, our study does not include PRH men treated in the island's healthcare system, affected by the downturn of the current PR economy.^31^ In addition, there is a possibility that a proportion of Hispanic men with PC in Continental US could present with similar poor outcomes as PRH patients introducing selection bias. We also must consider that our analysis approach could be underpowered to detect interactions. Finally, our findings in the VA system may not be generalizable to other clinical settings and practices.

In summary, our study suggests that in an equal access setting, PRH men treated with RP were at greater risk for BCR, metastases, CRPC, and PCSM during follow‐up despite having equal and better prognostic markers. Whether genetic background, comorbidity status, exposures, or other biological factors drive the significantly poorer PC outcomes in PR men is unknown.

## 5 CONCLUSIONS

Puerto Rican Hispanics patients after radical prostatectomy surgery generally had better pathological features, but despite this, they had significantly worse post‐treatment outcomes compared to men from the Continental US. Overall, the available evidence indicates that continued efforts are needed to understand PC among Hispanics, especially among men in Puerto Rico, who tend to have the worst outcomes.

## AUTHOR CONTRIBUTIONS

**Lourdes Guerrios‐Rivera:** Conceptualization (lead); investigation (equal); methodology (equal); resources (equal); validation (equal); visualization (equal); writing – original draft (lead); writing – review and editing (lead). **Jessica L. Janes:** Data curation (equal); formal analysis (lead); investigation (equal); methodology (equal); validation (equal); visualization (equal); writing – original draft (equal); writing – review and editing (equal). **Amanda M. De Hoedt:** Data curation (equal); investigation (equal); methodology (equal); project administration (lead); resources (equal); validation (equal); visualization (equal); writing – original draft (equal); writing – review and editing (equal). **Zachary Klaassen:** Data curation (equal); investigation (equal); resources (equal); validation (equal); writing – original draft (supporting); writing – review and editing (equal). **Martha K. Terris:** Conceptualization (equal); data curation (equal); investigation (equal); resources (equal); validation (equal); writing – original draft (supporting); writing – review and editing (equal). **Matthew R. Cooperberg:** Data curation (equal); investigation (equal); methodology (equal); resources (equal); validation (equal); visualization (equal); writing – original draft (supporting); writing – review and editing (equal). **Christopher L. Amling:** Conceptualization (equal); data curation (equal); investigation (equal); resources (equal); validation (equal); visualization (equal); writing – review and editing (equal). **Christopher J. Kane:** Conceptualization (equal); data curation (equal); investigation (equal); methodology (equal); resources (equal); validation (equal); visualization (equal); writing – review and editing (equal). **William J. Aronson:** Conceptualization (equal); data curation (equal); investigation (equal); methodology (equal); resources (equal); visualization (equal); writing – review and editing (equal). **Jay H. Fowke:** Conceptualization (equal); investigation (equal); methodology (equal); validation (equal); visualization (equal); writing – original draft (equal); writing – review and editing (equal). **Stephen J. Freedland:** Conceptualization (lead); data curation (lead); investigation (equal); methodology (equal); resources (equal); supervision (lead); validation (equal); visualization (equal); writing – original draft (equal); writing – review and editing (lead).

## FUNDING INFORMATION

The authors received no financial support for the research, authorship, or publication of this manuscript.

## CONFLICT OF INTEREST STATEMENT

All authors reported no conflict of interest.

## DISCLAIMER

The contents of this publication do not represent the views of the VA Caribbean Healthcare System, the Department of Veterans Affairs or the United States Government.

## Supporting information


Figure S1.

Table S1.

Table S2.

Table S3.

Table S4.

## Data Availability

Data are available upon request within VA rules and policy requirements.
