# Supplementary material for: Do Hispanic Puerto Rican men have worse outcomes after radical prostatectomy? Results from SEARCH
Source: Cancer Med. 2024 Mar 8;13(4):e7012. doi: 10.1002/cam4.7012 (PMC10922022; doi:10.1002/cam4.7012)

**Supplemental Figure 1. Kaplan-Meier survival curve for (A) Biochemical Recurrence (BCR), (B) metastasis, (C) Castrate-Resistant Prostate Cancer (CRPC), (D) Prostate Cancer Specific Mortality (PCSM) stratified by VA location (N = 8,311)**

A.


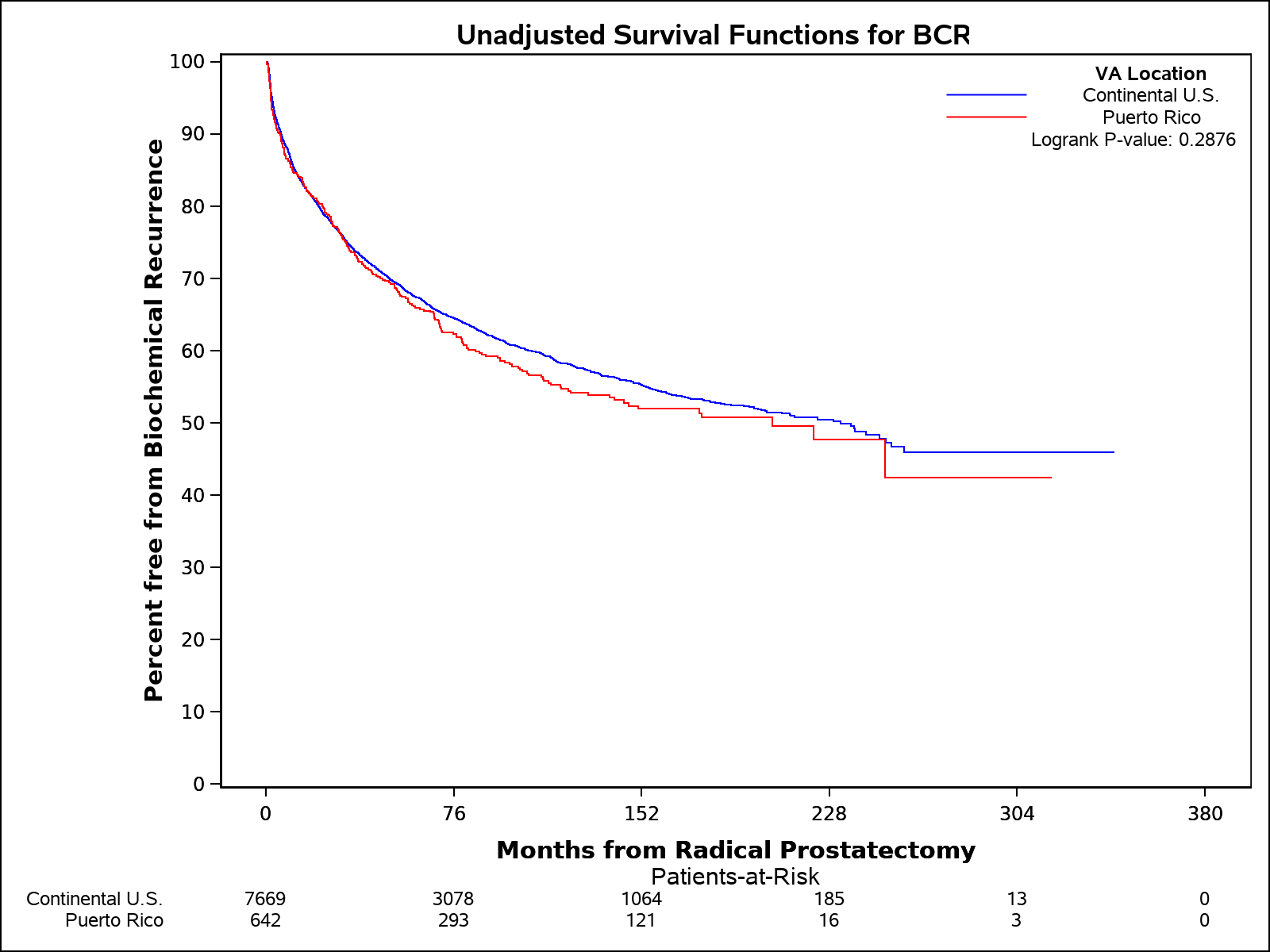


B.
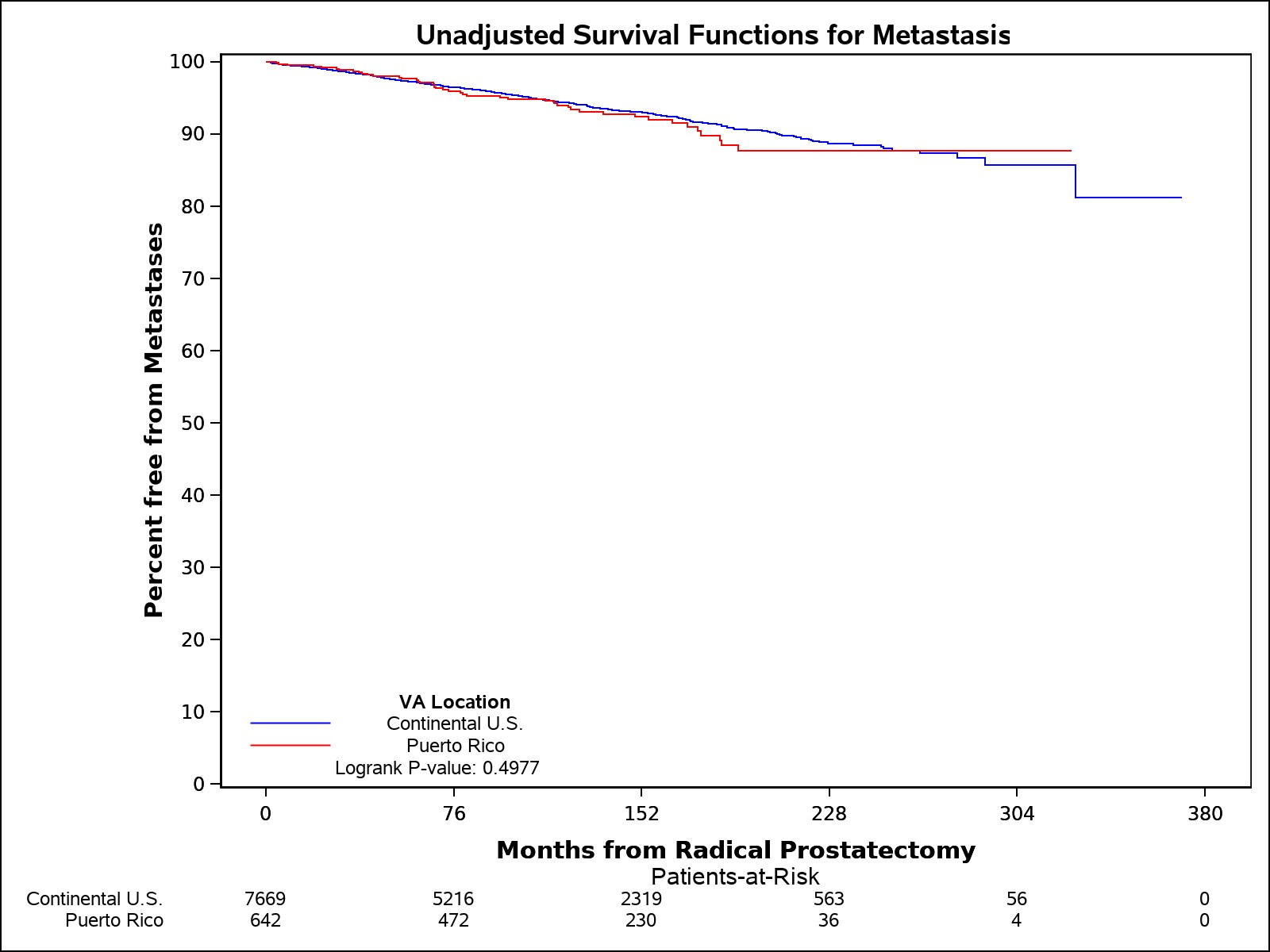


C.
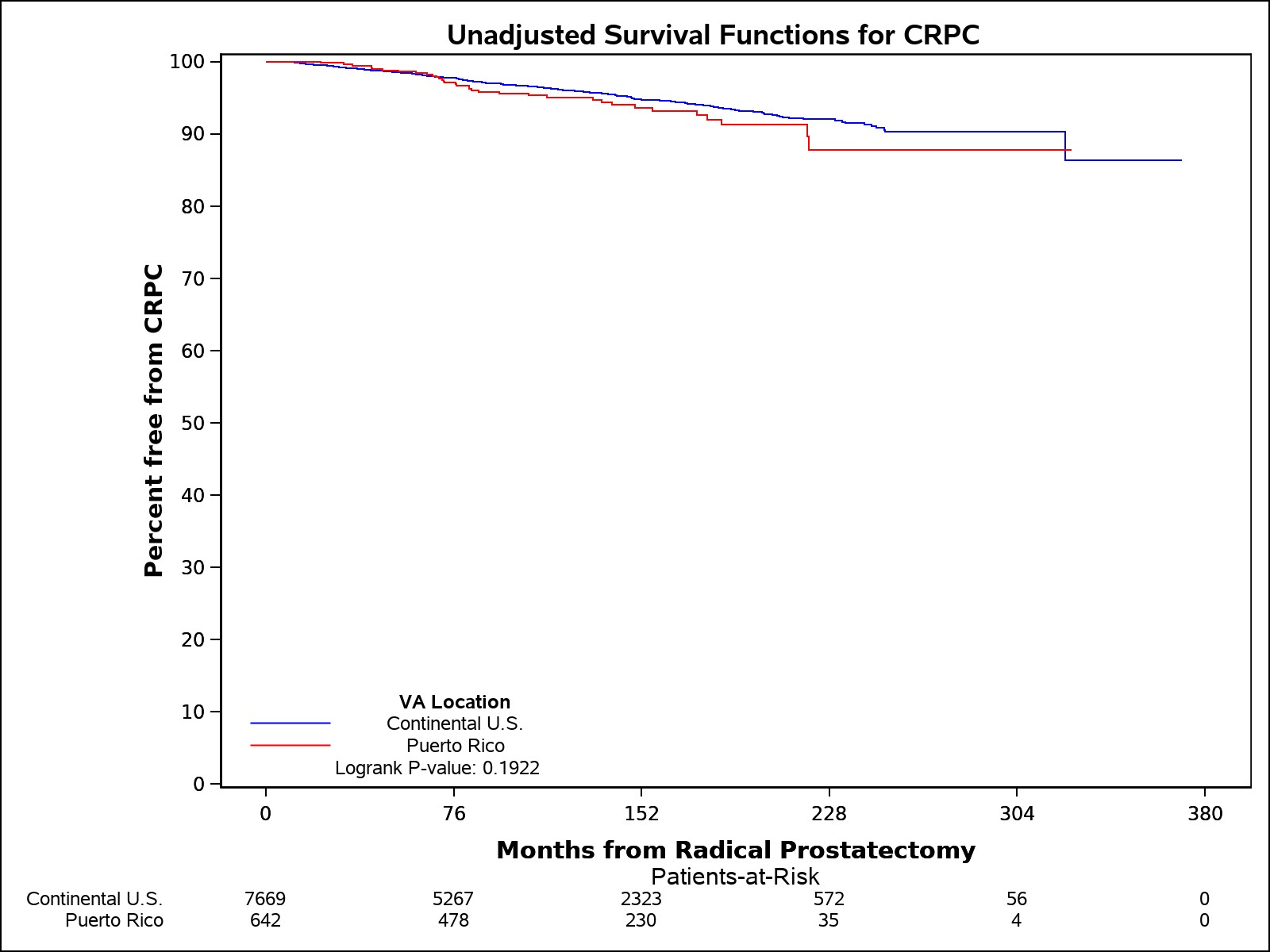


D.
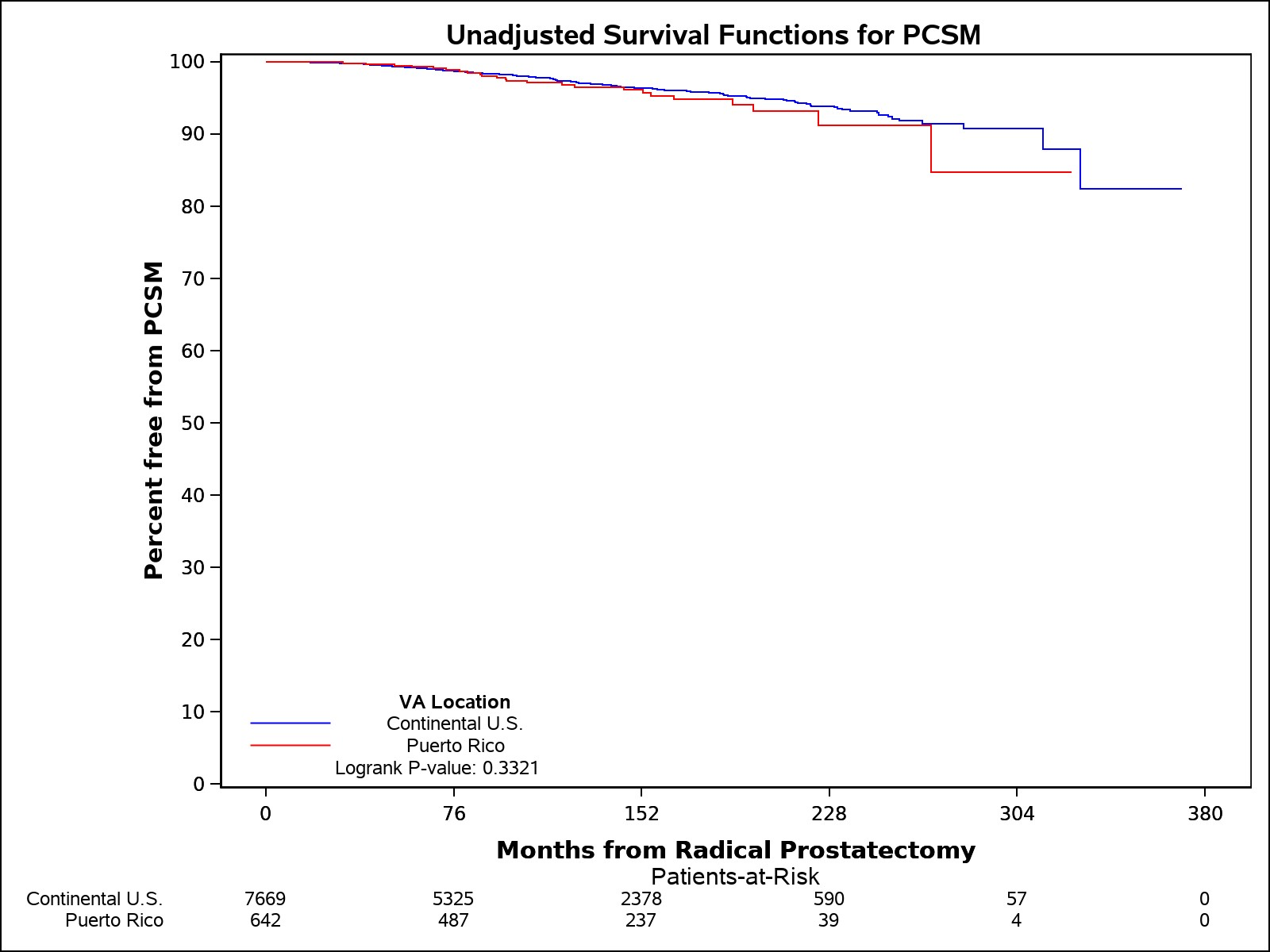

Supplement: Supplementary file 1 — Figure S1. [file CAM4-13-e7012-s003.docx]
