# Supplementary material for: Do Hispanic Puerto Rican men have worse outcomes after radical prostatectomy? Results from SEARCH
Source: Cancer Med. 2024 Mar 8;13(4):e7012. doi: 10.1002/cam4.7012 (PMC10922022; doi:10.1002/cam4.7012)
Supplement: Supplementary file 2 — Table S1. [file CAM4-13-e7012-s002.docx]

| **Supplemental Table 1. Odds of adverse pathological features estimated from multivariable logistic regression models (N = 8,311)** | | | | | | | | | | |
| --- | --- | --- | --- | --- | --- | --- | --- | --- | --- | --- |
|  | **Post-op Grade ≥ 2** | | **Lymph node metastasis**ꭞ | | **Positive surgical margins** | | **Seminal Vesicle Invasion** | | **Extracapsular extension** | |
| **Covariate** | **OR* (95% CI)** | **P value** | **OR* (95% CI)** | **P value** | **OR* (95% CI)** | **P value** | **OR* (95% CI)** | **P value** | **OR* (95% CI)** | **P value** |
|  |  |  |  |  |  |  |  |  |  |  |
| VA location |  | <0.001 |  | 0.001 |  | <0.001 |  | 0.089 |  | <0.001 |
| Continental US | Ref. |  | Ref. |  | Ref. |  | Ref. |  | Ref. |  |
| Puerto Rico | 0.52 (0.43, 0.63) |  | 0.25 (0.11, 0.57) |  | 0.43 (0.35, 0.53) |  | 0.75 (0.54, 1.04) |  | 1.55 (1.28, 1.87) |  |
| Age at surgery | 1.02 (1.01, 1.03) | 0.001 | 1.00 (0.98, 1.02) | 0.835 | 0.99 (0.99, 1.00) | 0.154 | 1.00 (0.99, 1.01) | 0.996 | 1.02 (1.01, 1.03) | <0.001 |
| Race |  | 0.002 |  | 0.313 |  | <0.001 |  | 0.026 |  | 0.006 |
| White | Ref. |  | Ref. |  | Ref. |  | Ref. |  | Ref. |  |
| Black | 1.25 (1.09, 1.43) |  | 0.82 (0.62, 1.10) |  | 1.25 (1.12, 1.38) |  | 1.19 (1.01, 1.40) |  | 0.82 (0.72, 0.92) |  |
| Other | 0.80 (0.57, 1.14) |  | 0.72 (0.35, 1.47) |  | 0.95 (0.73, 1.24) |  | 0.71 (0.44, 1.12) |  | 0.92 (0.68, 1.25) |  |
| Year of surgery | 1.11 (1.10, 1.12) | <0.001 | 1.04 (1.02, 1.06) | <0.001 | 0.99 (0.99, 1.00) | 0.161 | 0.99 (0.98, 1.00) | 0.144 | 1.01 (1.00, 1.01) | 0.167 |
| PSA (ng/mL) | 1.08 (1.07, 1.09) | <0.001 | 1.03 (1.02, 1.04) | <0.001 | 1.04 (1.04, 1.05) | <0.001 | 1.05 (1.04, 1.06) | <0.001 | 1.05 (1.04, 1.05) | <0.001 |
| Pre-op Grade |  | <0.001 |  | <0.001 |  | <0.001 |  | <0.001 |  | <0.001 |
| 1 | Ref. | <0.001 | Ref. |  | Ref. |  | Ref. |  | Ref. |  |
| 2 | 4.34 (3.77, 5.01) |  | 1.50 (0.94, 2.39) |  | 1.22 (1.08, 1.37) |  | 1.83 (1.47, 2.29) |  | 1.61 (1.39, 1.85) |  |
| 3 | 8.14 (6.32, 10.47) |  | 2.45 (1.52, 3.96) |  | 1.30 (1.12, 1.51) |  | 3.71 (2.91, 4.72) |  | 2.28 (1.92, 2.71) |  |
| 4-5 | 12.33 (9.50, 16.00) |  | 5.23 (3.40, 8.03) |  | 1.42 (1.23, 1.64) |  | 5.99 (4.82, 7.44) |  | 3.67 (3.14, 4.29) |  |
| Clinical stage |  | <0.001 |  | <0.001 |  | 0.125 |  | <0.001 |  | <0.001 |
| T1 | Ref. |  | Ref. |  | Ref. |  | Ref. |  | Ref. |  |
| T2 | 1.50 (1.33, 1.70) |  | 1.60 (1.25, 2.05) |  | 1.05 (0.95, 1.16) |  | 1.48 (1.27, 1.72) |  | 1.60 (1.44, 1.79) |  |
| T3/T4 | 1.87 (1.02, 3.43) |  | 3.59 (1.63, 7.91) |  | 1.55 (0.98, 2.45) |  | 5.15 (3.10, 8.55) |  | 6.49 (3.96, 10.63) |  |
| *Logistic regression models adjusted for VA location, biopsy Gleason grade, pre-operative PSA, year of surgery, age at surgery, race, and clinical stage.  ꭞModel for lymph node metastasis only among patients who had lymph node dissection done.  OR=Odds ratio, CI=Confidence Interval | | | | | | | | | | |
