# Supplementary material for: Do Hispanic Puerto Rican men have worse outcomes after radical prostatectomy? Results from SEARCH
Source: Cancer Med. 2024 Mar 8;13(4):e7012. doi: 10.1002/cam4.7012 (PMC10922022; doi:10.1002/cam4.7012)
Supplement: Supplementary file 3 — Table S2. [file CAM4-13-e7012-s001.docx]

| **Supplemental Table 2. Risk of long-term PC outcomes estimated from multivariable Cox proportional hazards models adjusted for pathological characteristics (N = 8,311)** | | | | | | | | |
| --- | --- | --- | --- | --- | --- | --- | --- | --- |
|  | **PCSM** | | **Metastasis** | | **CRPC** | | **BCR** | |
| **Covariate** | **HR (95% CI)** | **P value** | **HR (95% CI)** | **P value** | **HR (95% CI)** | **P value** | **HR (95% CI)** | **P value** |
| VA Location |  | 0.005 |  | 0.003 |  | <0.001 |  | <0.001 |
| Continental US | Ref. |  | Ref. |  | Ref. |  | Ref. |  |
| Puerto Rico | 1.89 (1.21, 2.95) |  | 1.64 (1.18, 2.26) |  | 1.90 (1.32, 2.75) |  | 1.51 (1.33, 1.72) |  |
| Post-op Grade |  | <0.001 |  | <0.001 |  | <0.001 |  | <0.001 |
| 1 | Ref. |  | Ref. |  | Ref. |  | Ref. |  |
| 2 | 1.79 (1.12, 2.86) |  | 1.89 (1.36, 2.62) |  | 1.57 (1.08, 2.30) |  | 1.65 (1.48, 1.84) |  |
| 3 | 2.83 (1.69, 4.76) |  | 3.23 (2.26, 4.62) |  | 2.58 (1.70, 3.93) |  | 2.38 (2.09, 2.70) |  |
| 4-5 | 6.63 (4.18, 10.51) |  | 6.40 (4.59, 8.92) |  | 5.93 (4.08, 8.63) |  | 2.94 (2.59, 3.34) |  |
| PSA (ng/mL) at surgery | 1.00 (0.99, 1.01) | 0.656 | 1.00 (0.99, 1.00) | 0.413 | 1.00 (0.99, 1.01) | 0.989 | 1.01 (1.01, 1.02) | <0.001 |
| Year of surgery | 0.95 (0.93, 0.97) | <0.001 | 0.99 (0.97, 1.00) | 0.046 | 0.96 (0.94, 0.98) | <0.001 | 0.99 (0.98, 0.99) | <0.001 |
| Age at surgery | 1.01 (0.99, 1.03) | 0.341 | 1.00 (0.99, 1.02) | 0.711 | 1.00 (0.98, 1.02) | 0.896 | 1.00 (0.99, 1.00) | 0.399 |
| Race |  | 0.719 |  | 0.623 |  | 0.265 |  | 0.001 |
| White | Ref. |  | Ref. |  | Ref. |  | Ref. |  |
| Black | 0.90 (0.65, 1.23) |  | 1.06 (0.86, 1.31) |  | 1.00 (0.77, 1.29) |  | 1.14 (1.05, 1.24) |  |
| Other | 0.82 (0.38, 1.75) |  | 0.81 (0.47, 1.41) |  | 0.51 (0.23, 1.15) |  | 1.26 (1.03, 1.54) |  |
| Extracapsular extension |  | <0.001 |  | <0.001 |  | <0.001 |  | <0.001 |
| No | Ref. |  | Ref. |  | Ref. |  | Ref. |  |
| Yes | 1.83 (1.37, 2.45) |  | 1.81 (1.47, 2.22) |  | 1.78 (1.39, 2.27) |  | 1.47 (1.35, 1.60) |  |
| Seminal vesicle invasion |  | <0.001 |  | <0.001 |  | <0.001 |  | <0.001 |
| No | Ref. |  | Ref. |  | Ref. |  | Ref. |  |
| Yes | 3.30 (2.45, 4.44) |  | 2.56 (2.07, 3.18) |  | 2.96 (2.30, 3.81) |  | 2.00 (1.81, 2.22) |  |
| Positive surgical margins |  | 0.230 |  | 0.245 |  | 0.521 |  | <0.001 |
| No | Ref. |  | Ref. |  | Ref. |  | Ref. |  |
| Yes | 1.19 (0.90, 1.57) |  | 1.12 (0.92, 1.37) |  | 1.08 (0.85, 1.37) |  | 1.98 (1.84, 2.14) |  |
| Lymph node metastasis |  | <0.001 |  | <0.001 |  | <0.001 |  | <0.001 |
| No | Ref. |  | Ref. |  | Ref. |  | Ref. |  |
| Not done | 0.80 (0.55, 1.17) |  | 0.76 (0.59, 0.99) |  | 0.73 (0.53, 1.01) |  | 0.89 (0.82, 0.98) |  |
| Yes | 2.14 (1.46, 3.15) |  | 1.73 (1.29, 2.31) |  | 2.33 (1.69, 3.21) |  | 1.45 (1.24, 1.69) |  |
| Models adjusted for VA location, pathological Gleason grade, pre-operative PSA, year of surgery, age at surgery, race, extracapsular extension, seminal vesicle invasion, surgical margins, and lymph node metastasis  HR = Hazard ratio, CI=Confidence Interval  PCSM = Prostate Cancer-Specific Mortality  CRPC = Castrate-Resistant Prostate Cancer  BCR = Biochemical Recurrence | | | | | | | | |
