# Supplementary material for: Do Hispanic Puerto Rican men have worse outcomes after radical prostatectomy? Results from SEARCH
Source: Cancer Med. 2024 Mar 8;13(4):e7012. doi: 10.1002/cam4.7012 (PMC10922022; doi:10.1002/cam4.7012)
Supplement: Supplementary file 4 — Table S3. [file CAM4-13-e7012-s004.docx]

| **Supplemental Table 3.** **Odds Ratios for VA Location stratified by biopsy grade group, clinical stage, and year of surgery** | | | | |
| --- | --- | --- | --- | --- |
| **Outcome** | **Group** | **OR** | **95% CI** | **Interaction p-value** |
| **Positive surgical margins** | **Puerto Rico vs Continental U.S. at pre-op Grade=1** | **0.28** | **(0.20, 0.39)** | **<0.001** |
|  | **Puerto Rico vs Continental U.S. at pre-op Grade =2** | **0.52** | **(0.36, 0.73)** |  |
|  | **Puerto Rico vs Continental U.S. at pre-op Grade=3** | **1.11** | **(0.59, 2.08)** |  |
|  | **Puerto Rico vs Continental U.S. at pre-op Grade= 4-5** | **0.61** | **(0.34, 1.07)** |  |
|  | Puerto Rico vs Continental U.S. at clinical stage=T1 | 0.41 | (0.30, 0.55) | 0.807 |
|  | Puerto Rico vs Continental U.S. at clinical stage=T2 | 0.45 | (0.33, 0.60) |  |
|  | Puerto Rico vs Continental U.S. at clinical stage=T3/T4 | 0.65 | (0.14, 2.97) |  |
|  | **Puerto Rico vs Continental U.S. at year=1994-2011** | **0.27** | **(0.20, 0.36)** | **<.0001** |
|  | **Puerto Rico vs Continental U.S. at year=<1994** | **0.76** | **(0.09, 6.31)** |  |
|  | **Puerto Rico vs Continental U.S. at year=>=2012** | **0.95** | **(0.69, 1.31)** |  |
| **Seminal vesicle invasion** | Puerto Rico vs Continental U.S. at pre-op Grade =1 | 1.03 | (0.61, 1.74) | 0.251 |
|  | Puerto Rico vs Continental U.S. at pre-op Grade =2 | 0.45 | (0.22, 0.91) |  |
|  | Puerto Rico vs Continental U.S. at pre-op Grade =3 | 0.57 | (0.21, 1.55) |  |
|  | Puerto Rico vs Continental U.S. at pre-op Grade =4-5 | 0.92 | (0.49, 1.73) |  |
|  | Puerto Rico vs Continental U.S. at clinical stage=T1 | 0.69 | (0.40, 1.18) | 0.926 |
|  | Puerto Rico vs Continental U.S. at clinical stage=T2 | 0.78 | (0.51, 1.20) |  |
|  | Puerto Rico vs Continental U.S. at clinical stage =T3/T4 | 0.84 | (0.15, 4.81) |  |
|  | Puerto Rico vs Continental U.S. at year=1994-2011 | 0.85 | (0.57, 1.27) | 0.685 |
|  | Puerto Rico vs Continental U.S. at year=<1994 | <0.001 | (0.00, 0.00) |  |
|  | Puerto Rico vs Continental U.S. at year=>=2012 | 0.63 | (0.35, 1.11) |  |
| **Extracapsular extension** | **Puerto Rico vs Continental U.S. at pre-op Grade =1** | **2.75** | **(2.12, 3.55)** | **<0.001** |
|  | **Puerto Rico vs Continental U.S. at pre-op Grade =2** | **0.93** | **(0.65, 1.32)** |  |
|  | **Puerto Rico vs Continental U.S. at pre-op Grade =3** | **0.79** | **(0.39, 1.59)** |  |
|  | **Puerto Rico vs Continental U.S. at pre-op Grade =4-5** | **0.93** | **(0.54, 1.60)** |  |
|  | Puerto Rico vs Continental U.S. at clinical stage =T1 | 1.95 | (1.49, 2.55) | 0.063 |
|  | Puerto Rico vs Continental U.S. at clinical stage =T2 | 1.25 | (0.96, 1.64) |  |
|  | Puerto Rico vs Continental U.S. at clinical stage =T3/T4 | 2.27 | (0.41, 12.55) |  |
|  | **Puerto Rico vs Continental U.S. at year=1994-2011** | **2.62** | **(2.10, 3.3)** | **<.0001** |
|  | **Puerto Rico vs Continental U.S. at year=<1994** | **15.99** | **(1.63, 157.00)** |  |
|  | **Puerto Rico vs Continental U.S. at year=>=2012** | **0.35** | **(0.22, 0.54)** |  |
| **Lymph node metastasis** | Puerto Rico vs Continental U.S. at pre-op Grade =1 | <0.001 | (0.00, 0.00) | 0.894 |
|  | Puerto Rico vs Continental U.S. at pre-op Grade =2 | 0.47 | (0.14, 1.52) |  |
|  | Puerto Rico vs Continental U.S. at pre-op Grade =3 | 0.30 | (0.04, 2.29) |  |
|  | Puerto Rico vs Continental U.S. at pre-op Grade =4-5 | 0.23 | (0.05, 0.96) |  |
|  | Puerto Rico vs Continental U.S. at clinical stage=T1 | 0.22 | (0.05, 0.94) | 0.958 |
|  | Puerto Rico vs Continental U.S. at clinical stage =T2 | 0.29 | (0.11, 0.80) |  |
|  | Puerto Rico vs Continental U.S. at clinical stage =T3/T4 | <0.001 | (0.00, 0.00) |  |
|  | Puerto Rico vs Continental U.S. at year=1994-2011 | 0.19 | (0.05, 0.76) | 0.806 |
|  | Puerto Rico vs Continental U.S. at year=<1994 | <0.001 | (0.00, 0.00) |  |
|  | Puerto Rico vs Continental U.S. at year=>=2012 | 0.33 | (0.12, 0.93) |  |
| **Post-op Grade ≥ 2** | Puerto Rico vs Continental U.S. at pre-op Grade =1 | 0.59 | (0.47, 0.76) | 0.834 |
|  | Puerto Rico vs Continental U.S. at pre-op Grade =2 | 0.60 | (0.40, 0.90) |  |
|  | Puerto Rico vs Continental U.S. at pre-op Grade =3 | 0.57 | (0.19, 1.71) |  |
|  | Puerto Rico vs Continental U.S. at pre-op Grade =4-5 | 1.06 | (0.32, 3.53) |  |
|  | Puerto Rico vs Continental U.S. at clinical stage =T1 | 0.67 | (0.51, 0.88) | 0.548 |
|  | Puerto Rico vs Continental U.S. at clinical stage =T2 | 0.54 | (0.40, 0.72) |  |
|  | Puerto Rico vs Continental U.S. at clinical stage =T3/T4 | 0.76 | (0.13, 4.59) |  |
|  | Puerto Rico vs Continental U.S. at year=1994-2011 | 0.67 | (0.54, 0.83) | 0.087 |
|  | Puerto Rico vs Continental U.S. at year=<1994 | 0.69 | (0.07, 6.69) |  |
|  | Puerto Rico vs Continental U.S. at year=>=2012 | 0.38 | (0.24, 0.60) |  |
