# Supplementary material for: Do Hispanic Puerto Rican men have worse outcomes after radical prostatectomy? Results from SEARCH
Source: Cancer Med. 2024 Mar 8;13(4):e7012. doi: 10.1002/cam4.7012 (PMC10922022; doi:10.1002/cam4.7012)
Supplement: Supplementary file 5 — Table S4. [file CAM4-13-e7012-s005.docx]

| **Supplemental Table 4. Hazard Ratios for VA Location stratified by biopsy grade group, clinical stage, and year of surgery** | | | | | |
| --- | --- | --- | --- | --- | --- |
| **Outcome** | **Group** | **HR** | **95% CI** | | **Interaction p-value** |
| **PCSM** | Puerto Rico vs Continental U.S. at pre-op Grade=1 | 2.51 | 1.28 | 4.92 | 0.457 |
|  | Puerto Rico vs Continental U.S. At pre-op Grade=2 | 1.25 | 0.50 | 3.15 |  |
|  | Puerto Rico vs Continental U.S. At pre-op Grade=3 | 2.17 | 0.51 | 9.14 |  |
|  | Puerto Rico vs Continental U.S. At pre-op Grade=4-5 | 1.17 | 0.51 | 2.68 |  |
|  | Puerto Rico vs Continental U.S. At year<1994 | 0.00 | 0.00 | 0.00 | 0.716 |
|  | Puerto Rico vs Continental U.S. At year=1994-2011 | 1.78 | 1.15 | 2.77 |  |
|  | Puerto Rico vs Continental U.S. At year>=2012 | 0.76 | 0.10 | 5.63 |  |
|  | Puerto Rico vs Continental U.S. At clinical stage=T1 | 1.51 | 0.72 | 3.15 | 0.914 |
|  | Puerto Rico vs Continental U.S. At clinical stage=T2 | 1.83 | 1.08 | 3.09 |  |
|  | Puerto Rico vs Continental U.S. At clinical stage=T3/T4 | 0.00 | 0.00 | 0.00 |  |
| **Metastasis** | **Puerto Rico vs Continental U.S. at pre-op Grade=1** | **2.61** | **1.62** | **4.22** | **0.042** |
|  | **Puerto Rico vs Continental U.S. At pre-op Grade=2** | **1.18** | **0.61** | **2.26** |  |
|  | **Puerto Rico vs Continental U.S. At pre-op Grade=3** | **0.69** | **0.17** | **2.80** |  |
|  | **Puerto Rico vs Continental U.S. At pre-op Grade=4-5** | **1.02** | **0.54** | **1.93** |  |
|  | Puerto Rico vs Continental U.S. At year<1994 | 0.00 | 0.00 | 0.00 | 0.523 |
|  | Puerto Rico vs Continental U.S. At year=1994-2011 | 1.60 | 1.15 | 2.23 |  |
|  | Puerto Rico vs Continental U.S. At year>=2012 | 0.87 | 0.32 | 2.37 |  |
|  | Puerto Rico vs Continental U.S. At clinical stage=T1 | 1.37 | 0.82 | 2.31 | 0.913 |
|  | Puerto Rico vs Continental U.S. At clinical stage=T2 | 1.58 | 1.07 | 2.34 |  |
|  | Puerto Rico vs Continental U.S. At clinical stage=T3/T4 | 0.00 | 0.00 | 0.00 |  |
| **CRPC** | Puerto Rico vs Continental U.S. at pre-op Grade=1 | 2.59 | 1.48 | 4.54 | 0.344 |
|  | Puerto Rico vs Continental U.S. At pre-op Grade=2 | 1.20 | 0.52 | 2.78 |  |
|  | Puerto Rico vs Continental U.S. At pre-op Grade=3 | 1.11 | 0.27 | 4.57 |  |
|  | Puerto Rico vs Continental U.S. At pre-op Grade=4-5 | 1.51 | 0.81 | 2.78 |  |
|  | Puerto Rico vs Continental U.S. At year<1994 | 0.00 | 0.00 | 0.00 | 0.499 |
|  | Puerto Rico vs Continental U.S. At year=1994-2011 | 1.88 | 1.30 | 2.72 |  |
|  | Puerto Rico vs Continental U.S. At year>=2012 | 0.78 | 0.19 | 3.23 |  |
|  | Puerto Rico vs Continental U.S. At clinical stage=T1 | 1.11 | 0.560 | 2.20 | 0.255 |
|  | Puerto Rico vs Continental U.S. At clinical stage=T2 | 2.18 | 1.44 | 3.29 |  |
|  | Puerto Rico vs Continental U.S. At clinical stage=T3/T4 | 0.00 | 0.00 | 0.00 |  |
| **BCR** | Puerto Rico vs Continental U.S. at pre-op Grade=1 | 1.26 | 1.04 | 1.52 | 0.820 |
|  | Puerto Rico vs Continental U.S. At pre-op Grade=2 | 1.35 | 1.08 | 1.70 |  |
|  | Puerto Rico vs Continental U.S. At pre-op Grade=3 | 1.08 | 0.67 | 1.73 |  |
|  | Puerto Rico vs Continental U.S. At pre-op Grade=4-5 | 1.19 | 0.87 | 1.62 |  |
|  | Puerto Rico vs Continental U.S. At year<1994 | 1.29 | 0.41 | 4.06 | 0.677 |
|  | Puerto Rico vs Continental U.S. At year=1994-2011 | 1.22 | 1.05 | 1.41 |  |
|  | Puerto Rico vs Continental U.S. At year>=2012 | 1.39 | 1.08 | 1.78 |  |
|  | Puerto Rico vs Continental U.S. At clinical stage=T1 | 1.36 | 1.13 | 1.64 | 0.486 |
|  | Puerto Rico vs Continental U.S. At clinical stage=T2 | 1.17 | 0.99 | 1.40 |  |
|  | Puerto Rico vs Continental U.S. At clinical stage=T3/T4 | 1.50 | 0.60 | 3.77 |  |
